# Low-Pass Filter for HV Partial Discharge Testing

**DOI:** 10.3390/s18020482

**Published:** 2018-02-06

**Authors:** Vladimir Kindl, Bohumil Skala, Roman Pechanek, Vaclav Kus, Jaroslav Hornak

**Affiliations:** University of West Bohemia, Faculty of Electrical Engineering, Regional Innovation Centre for Electrical Engineering (RICE), Univerzitni 8, 301 00 Pilsen, Czech Republic; skalab@kev.zcu.cz (B.S.), rpechane@kev.zcu.cz (R.P.), kus@kev.zcu.cz (V.K.); jhornak@ket.zcu.cz (J.H.)

**Keywords:** low-pass filter, partial discharge, design, magnetic field, finite element method

## Abstract

The most common cause of high voltage electric machine malfunction is an electrical failure of the insulation system due to extreme partial discharges activity. The paper discusses the methodology for the construction of a low-pass high voltage filter for partial discharge measurement. It focuses mainly on the shape optimization, using an analytical approach with subsequent verification using the finite element method. The experimental verification is given together with important conclusions.

## 1. Introduction

Nowadays, industry is virtually dependent on a continuous supply of electric energy. The most important component of the ordinary power plant is a power generator, but the plant normally utilizes a large amount of supporting drives and auxiliary systems, which together forms a very complicated functional unit. Every electrical device operating in such a system is inherently at risk of failure due to many external or internal matters (e.g., excessive electrical, thermal, or mechanical stress, extreme operating conditions, etc.) [1,2,3,4], and even a brief drop-out of any auxiliary system may lead to incidental power plant downtime, and thus power outage. Therefore, machine reliability and availability are in high demand. Many studies [5,6,7] have reported that one of the most common causes of high voltage electric machine malfunction is an electrical failure of the insulation system due to extreme partial discharges (PD) activity.

In such a case, the insulating system is subjected to several stresses that cause changes of material properties. This effect progressively reduces the ability of the insulation to resist even normal operational stresses. Precise determination of the actual condition of the insulating system may prevent its destructive changes and help in planning lay-offs or regular maintenance.

**Figure 1:**
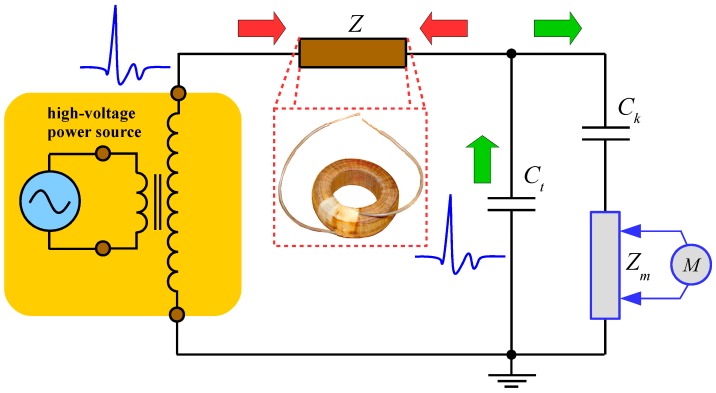
Basic partial discharges (PD) measurement test circuit [17].

Therefore, the detection and evaluation of PD activity [8,9,10,11,12,13] is a very important issue for systems reliability. Nowadays, PD detection [14,15,16,17,18,19,20] as a non-destructive diagnostic tool is used in a wide range of applications, e.g., in generators, transformers, cables, switchgears, and many others. One of the most common test circuits of the galvanic PD measurement method (according to International Electrotechnical Commission standard IEC 60270 [21]) is illustrated in Figure 1.

The measuring circuit is powered by a high voltage source feeding two branches though the filter. The first one involves the tested object *C_t_*, in which PD occurs. The second one consists of a capacitive divider formed by a coupling capacitor *C_k_* and an impedance *Z_m_*, which converts the high frequency current to a voltage signal sensed by the measuring system *M*. The filter *Z* must be connected to the source output to prevent any noise and interference coming from the power supply site. The required PD signal will remain in PD measuring loop and will not leak through the power supply to ground. The filter is usually built as a large inductor because the tested insulation system *C_t_* exhibits a predominantly capacitive character.

**Figure 2:**
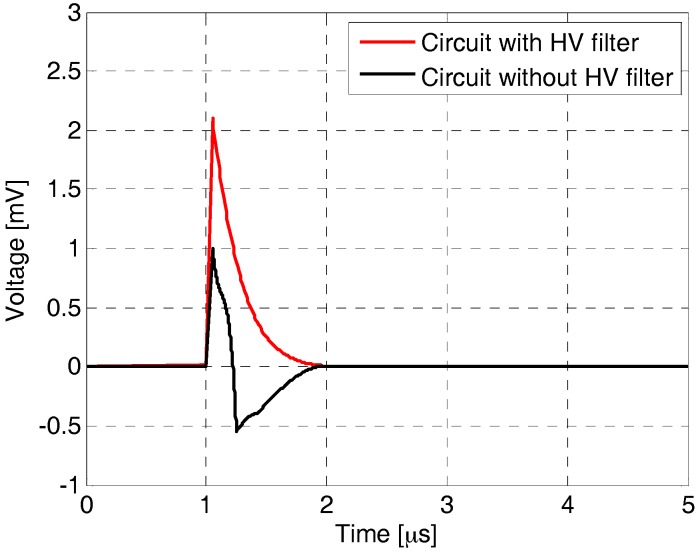
Calibrating signal measured in the testing circuit (Figure 1) supplied by a 30-m-long shielded cable.

The high voltage (HV) filter plays an important role, especially in the diagnostics of rotary machines and transformers that are fed through an HV cable. Due to the long power supply cable, the measured signal may contain additional oscillations, reflections, and attenuation. The typical calibration pulse used in an HV circuit for PD calibration and its variation with and without a filter is presented in Figure 2.

This article proposes a design method for an HV filter applicable for PD measurement. The filter is constructed as an air-cored coil with defined inductance and optimized parasitic capacitance to meet a high-quality factor and predefined self-resonance frequency. The filter can be considered as a complicated RLC circuit (see Figure 8) with complex mutual interconnections. When the inductance coils predefine the resonance frequency according to the tested insulation system, the parasitic capacitance affects its attenuation.

Therefore, many applications require the lowest stray capacitance possible. However, in this particular case, it is desired to set the stray capacitance so it causes the self-resonance frequency of the filter to be between 80 kHz and 140 kHz.

## 2. Filter Design and Basic Specification

In the past, the design process was limited due to the low available computational performance of common computers. So, it was necessary to produce a number of prototypes showing the proper way of construction. This was time-consuming and resulted in additional financial and material losses. However, the modern design method, usually called virtual prototyping, considers many technical requirements and electrical and space limitations and therefore reduces the time and money spent during the development. The virtual prototyping used in this approach includes an analytical design with subsequent verification via finite element analyses (FEA). The FEA includes both the magnetic field and thermal analyses. The proposed design methodology is demonstrated on an HV filter with the following technical requirements, as seen in Table 1.

**Figure 3:**
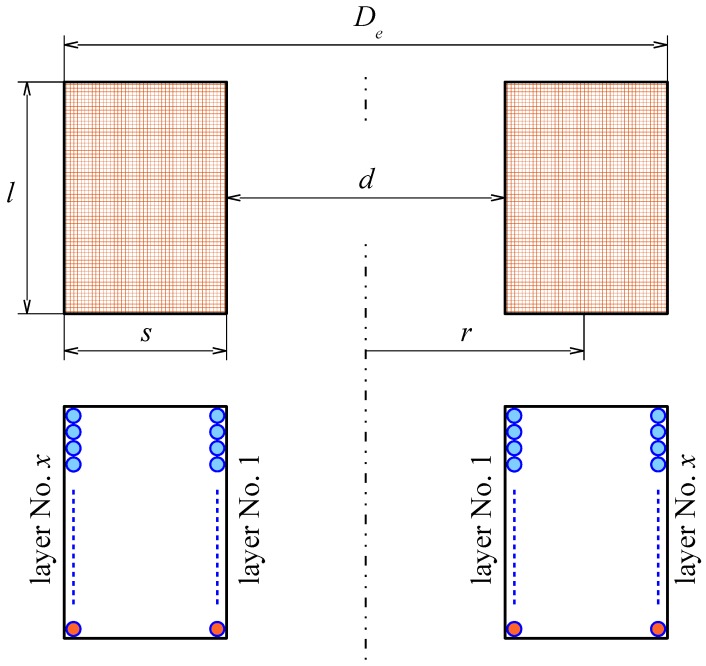
Basic design outline of the filter.

First, the main dimensions (inner and outer diameter, length of the winding and number of turns, see Figure 3) can be calculated using simple analytical formulas, detailed here as Equations (1)–(7) [22,23].

Wheeler’s formula [24] determines the coil’s inductance for the expected geometry as Equation (1).

(1) $$L=3.15\;\times \;10^{-5}\frac{N^{2}r^{2}}{6r+9l+10s}$$

The current density *J* and the filling coefficient of the winding *k* may be derived from (2) by using the rated current *I*, conductor cross-section area *S_Cu_*, and number of turns *N*. The parameter *V_Cu_* represents the total volume of the used wire.

(2) $$J=\frac{I}{S_{Cu}},\;k=\frac{NS_{Cu}}{sl}\;=\;\frac{V_{Cu}}{V}=\frac{l_{Cu}S_{Cu}}{2\pi rsl}$$

The winding volume *V* is a product of the total length of the wire *l_Cu_, k* and the wire cross-section area *S_Cu_,* and hence, taking Equation (2) into consideration, we may derive the central radius *r* from Equation (3).

(3) $$r=\frac{V_{Cu}}{2\pi sl}=\frac{l_{Cu}S_{Cu}}{2\pi ksl}$$

Consequently, Equation (1) can be rewritten as a function of only two variables (*s* and *l*), as given by Equation (4).

(4) $$L\left(s,l\right)=\frac{3.15\;\times \;10^{-5}}{2\pi }\frac{kl_{Cu}^{2}sl}{6l_{Cu}S_{Cu}+18\pi ksl^{2}+20\pi kls^{2}}$$

The maximum of this function may be found from the solution of the partial derivative of Equation (4) compared to zero. This results in the determination of *s* and *l*, as given in Equation (5), for which Equation (4) reaches its maximum.

(5) $$s=0.441\sqrt[{3}]{\frac{l_{Cu}S_{Cu}}{k}},\;l=0.490\sqrt[{3}]{\frac{l_{Cu}S_{Cu}}{k}}$$

It is further possible to manipulate the model in order to obtain any desired dimension, e.g., *r*, *d*, or *D_e_.*

(6) $$r=0.736\sqrt[{3}]{\frac{l_{Cu}S_{Cu}}{k}},\;d=2\left(r-\frac{s}{2}\right),\;D_{e}=2\left(r+\frac{s}{2}\right)$$

Additionally, the number of turns is given by Equation (7).

(7) $$N=167.2\;\sqrt[{5}]{\frac{J\pi L^{2}}{4I}}$$

If we consider *d* to be a determining dimension, then we obtain the geometry as follows: outer diameter *D_e_* = 1.855 × *d*, central radius *r* = 0.714 × *d*, height of the coil *l* = 0.475 × *d*, and its width *s* = 0.428 × *d*. These values correspond to the optimized design which maximizes the inductance and minimizes the copper volume. The proposed model makes the optimization very quick and easy and may be used in various ways. Another approach is given, for example, in Reference [25].

The resulting design has winding made from copper wire with a diameter of 1.9 mm assembled into 12 layers, with each having 18 turns. The total number of turns is therefore *N* = 12 × 18 = 216. Other important design properties are given in Table 2.

**Figure 4:**
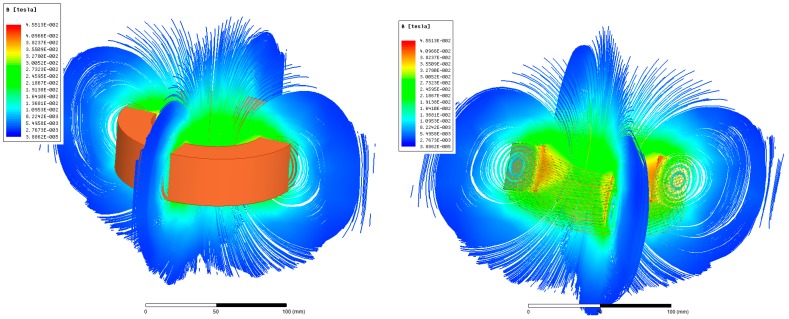
Finite element analyses (FEA) made under selected designs: magnetic flux density (**left**); magnetic flux density including current density in the winding (**right**).

While the analytical approach is very general, fast, transparent, and informative, its ability to consider complex geometry is very limited. This may cause unexpected and inestimable error in obtained results. Therefore, it is necessary to verify the given geometry by using some other method. FEA was used in this case (see Figure 4). The winding is modeled as a hollow cylinder with a defined number of turns. No eddy currents are included and therefore the simulation neglects both the skin-effect and the proximity-effect. Taking into account the low simulation supplying frequency, the error caused by the simplification is negligible.

The coil’s inductance, derived from the magnetic energy, stored in all electromagnetic regions, is equal to 6.15 mH. The rated current generates 72.8 W of I^2^R losses.

## 3. Thermal Analysis

The filter is designed to be capable of self-cooling. The category IC410 (surface cooled by natural convection and radiation) with no external fan is required to make the system robust, reliable, and low-cost. In this case, the heat is transferred from the winding to the insulation varnish due to heat conduction, then it goes to the coil frame from which it is removed using convection to the coolant (air). The outer surface is also cooled by heat convection. The heat transfer coefficient [26] for the outer surface and estimated surface temperature rice *ϑ* is given by Equation (8).

(8) $$\alpha =11.3+0.08\vartheta \left[\frac{W}{m^{2}K}\right]$$

For the inner surface, a similar formula (Equation (9)) takes place. The heat radiation can be neglected.

(9) $$\alpha =6.5+0.05\vartheta \left[\frac{W}{m^{2}K}\right],\;\mathrm{for}\;\vartheta \in \langle 0|100\rangle {}^{\circ}C$$

**Figure 5:**
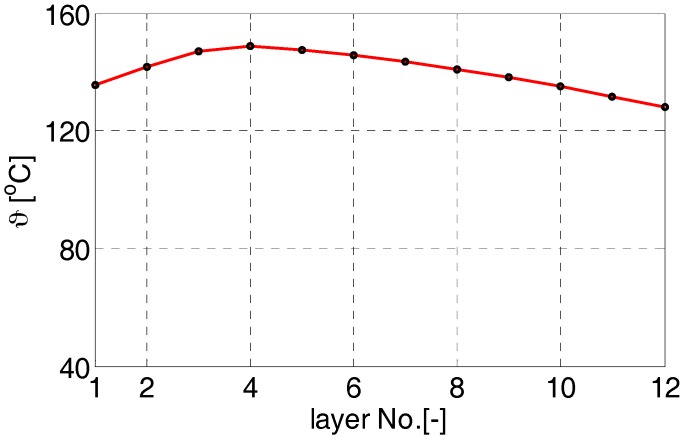
Steady-state temperature distribution in the winding estimated for the full-load operation.

The insulating system is classified in the temperature thermal class “H” (180 °C), and because no temperature sensor is installed in it, further temperature evaluation may be done using a direct current test (the four-terminal sensing method). The calculations of Equations (8)–(10) were performed, focusing on the heat transfer and the system cooling ability. The model is valid for those coil turns located in the middle of each winding layer. The turns close to the coil side (close to the coil frame) have a significantly lower temperature. Moreover, the heat transfer in the simulation is considered to be a 2D (not a 3D) vector, which means that the worst-case scenario is investigated (see Figure 5). The thermal conductivity of the coil form slightly increases the inner surface temperature with respect to the outer surface temperature. The power losses Δ*P_i_* of the *i*-th winding layer are given by Equation (10).

(10) $$\Delta P_{i}=R_{i}I^{2}\left[W\right]$$

The area of the cylinder’s surface related to the *i*-th winding layer can be calculated using Equation (11).

(11) $$S_{i}=\pi d_{i}l\left[m^{2}\right]$$

The temperature difference of the *i*-th winding layer is then given by Equation (12).

(12) $$\Delta \vartheta_{i}=\frac{\Delta P_{i}}{\alpha S_{i}}\left[K\right]$$

As seen in Figure 5, the maximal temperature reaches 149 °C, whereas the inner and outer surface temperatures are cooler (136/128 °C). Ambient temperature is assumed as a constant of 40 °C. The results correspond to the stabilized full-load operation.

The quick analytical thermal analyses show potentially good operability with no problematic hotpots or possible thermal degradation of the insulating system. Hence, we may also verify the design using FEA.

**Figure 6:**
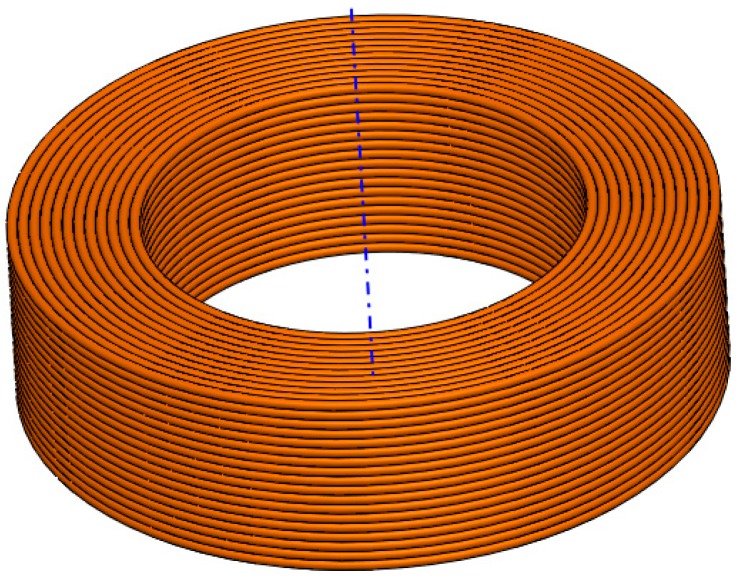
Detailed model of the filter considering the real geometry of the winding.

The coil model seen in Figure 6 is built using ANSYS software. It fully corresponds to the investigated geometry and design parameters listed in Table 2.

**Figure 7:**
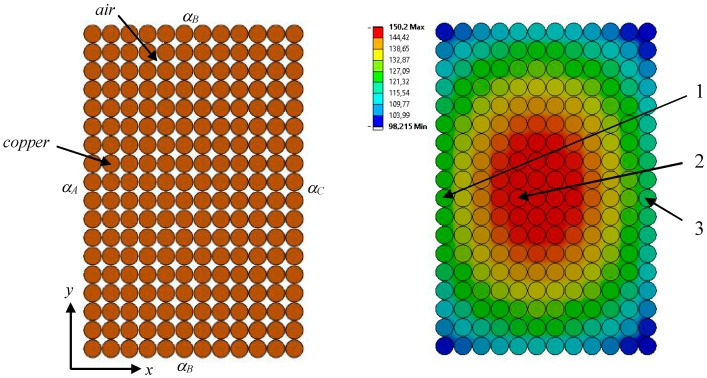
Model boundary condition settings (**left**); temperature distribution in the coil cross-section (**right**).

The thermal model is represented by the two materials. The copper represents the coil wires and the air fills the empty space between wires. The insulation thickness on the outer diameter of each wire is 0.03 mm. It is modeled through contact regions with fictive heat resistance. A detailed model with a description of the boundary condition and material settings is given in left side of Figure 7. The mathematical description [27,28] for thermal FEA is summarized in Equation (13).

(13) $$𝛻\left(\lambda \left(\vartheta \right)𝛻\vartheta \right)-\rho \left(\vartheta \right)c_{p}\left(\vartheta \right)\frac{\partial \vartheta }{\partial t}=-p_{j},$$

where λ is the thermal conductivity, ϑ represents the temperature, ρ symbolizes mass density, and *c_p_* stands for the specific heat. As shown below, the right side in Equation (8) represents the heat losses generated in the coil wires, given by Equation (14).

(14) $$p_{j}=\frac{{\left\|J\right\|}^{2}}{\gamma }$$

The parameter γ is the electric conductivity of the relevant material and, hence, the heat loss stored in the air is equal to zero. Boundary conditions between respective wires and air are predefined by the constant heat flow, as shown by Equation (15).

(15) $${\lambda \frac{\partial \vartheta }{\partial t}|}_{Cu}={\lambda \frac{\partial \vartheta }{\partial t}|}_{Air}$$

We may set the mixed boundary conditions (Equation (16)) for the coil outer surfaces and the ambient air. Here, the heat transfer coefficients $\alpha_{A,B,C}$ correspond to Equations (8) and (9) (see Figure 7).

(16) $${-\lambda \frac{\partial \vartheta }{\partial t}|}_{Coil}={\alpha_{A,B,C}\left(\vartheta \right)\left(\vartheta -\vartheta_{Amb}\right)|}_{Amb}\;{\alpha_{A,B}|}_{9}\;{\alpha_{C}|}_{8}$$

The steady-state temperature distribution determined by FEA is shown in the right side of Figure 7. The highest temperature (150 °C) is measured at the wire marked with number 2. The hottest wire at the coil’s inner diameter (the turn marked with number 1) has a temperature of 127 °C. The hotspot at the outer diameter (number 3) has a temperature of 120 °C. The temperature difference recorded between FEA and the analytical approach is less than 8%.

## 4. Winding Parasitic Capacitance

**Figure 8:**
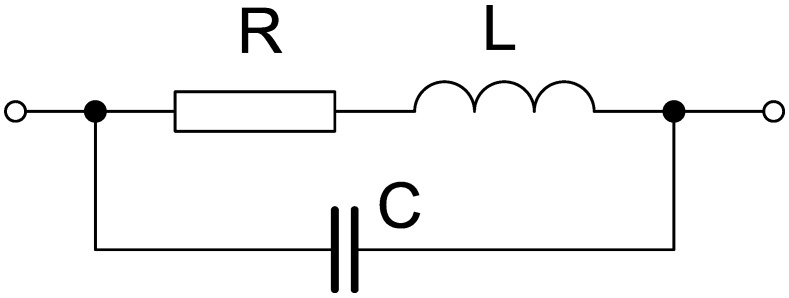
Equivalent electrical circuit for a coil.

Any electric field present between coil turns forms a usually unwanted stray capacitance of the coil (see Figure 8). This capacitance acts contrary to the self-inductance and modifies the operational quality factor. Hence, it is desirable to reduce it as much as possible. In this particular case, we need to set it so it causes the self-resonance frequency of the filter to be somewhere between 80 kHz and 140 kHz.

**Figure 9:**
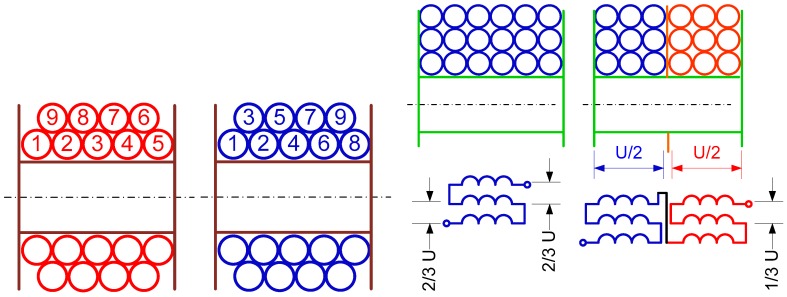
Winding with no transposition (red) and transposed winding (blue) reducing the stray capacitance (**left-side** figure); winding divided into sections (**right-side** figure).

In general, the stray capacitance could be manipulated by using a special design (transposition) of the winding. The transposition increases the mutual distance between certain neighboring wires, which consequently reduces the resulting stray capacitance. This situation can be seen in the left side of Figure 9.

**Figure 10:**
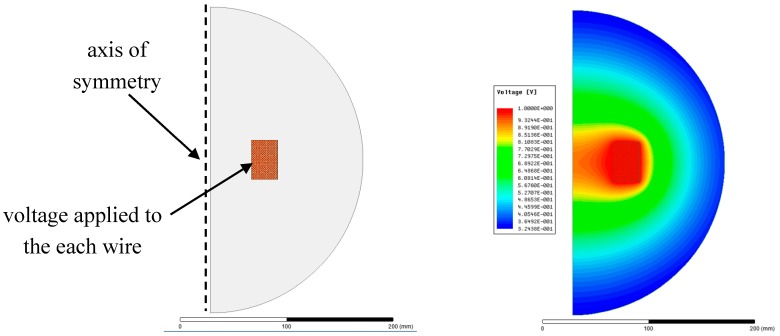
Axisymmetric FE model (**left**); calculated voltage distribution (**right**) across the model.

Another possibility is to divide the winding into several sections, as depicted in the right side of Figure 9, and connect them in series. This method reduces the winding stray capacitance due to the lower voltage present between turns. It is not as effective as the transposed winding, but sometimes it finds its purpose. For the discussed filter, we used transposed winding, as described, as a first case. The matrix of stray capacitances formed between the respective coil turns can be calculated using electrostatic FEA. In that case, the usage of the axisymmetric 2D model shown in Figure 10 is beneficial.

**Figure 11:**
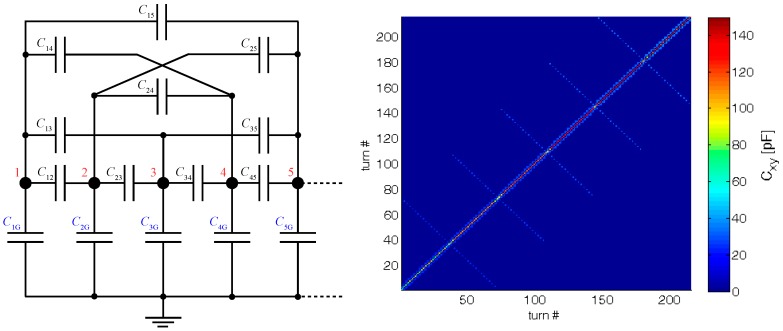
Equivalent model (node-to-node lumped capacitance network) for the first five turns of the winding (**left**); complete capacitance matrix expressed as a 3D plot (**right**).

The voltage applied to the winding in the simulation has no physical connection with the real operation, but it is only used for the extraction of the stray capacitance matrix. Table 3 presents the calculated parasitic capacitances of the first five coil turns. The value of the particular capacitance is determined by a combination of a certain matrix row and column. For example, the parasitic capacitance between the second and the third turn of the coil (*C*_23_ = *C*_32_) appears at the cell located either at the intersection of the second column with the third row (colored cells in the Table 3), or at the intersection of the second row with the third column. Both values are the same, *C*_23_ = *C*_32_ = 2.1 pF, and therefore the matrix is symmetrical. The values written in the main diagonal represent the wire-ground capacitance (see Figure 11).

The distributed capacitance of an inductor is modeled by a network of lumped node-to-node capacitance elements, as given in Figure 11, where each node represents a turn.

The calculation of the net capacitance by a 2D electrostatic axisymmetric FEA is very fast, especially when an inductor with a very small number of turns is analyzed. However, this specific filter requires relatively long post-processing. The net capacitance determined using FEA is 226 pF.

## 5. Experiments

First, the temperature-rise test was made to determine if the filter is able to operate under the rated load. As mentioned before, the filter has no thermal sensor implemented in the winding, and therefore the temperature must be determined from the DC winding resistance. We applied the four-terminal sensing method (Kelvin resistance measurement) for this purpose, since it provides us with quite accurate results.

**Figure 12:**
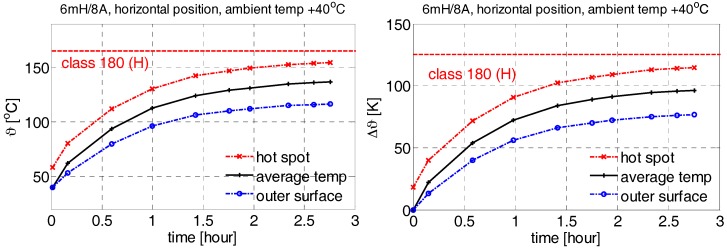
Temperature rise in the filter under full load operation (**left**); temperature difference (**right**).

As seen from Figure 12, all the temperatures slightly rise with the loading time (input current constant), whereas the maximal value of the temperature lies safely under the limit of the winding insulation class. The red line with cross markers represents the calculated temperature inside the winding (hot spot), the black curve with “+” markers is the average temperature measured from the winding resistance, and the blue line with circle markers represents the temperature measured on the outer surface with a thermometer.

The self-inductance of the filter was measured with an LCR meter. The measured value (6.05 mH) fits the calculated value very closely.

**Figure 13:**
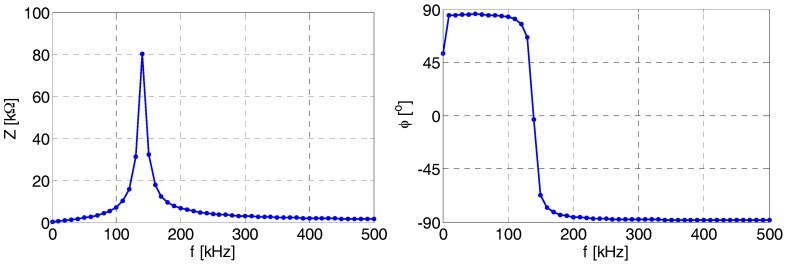
Frequency-dependent net impedance of the filter (**left**) and the phase shift measured between the supplying voltage and the input current (**right**).

Then, we analyzed the self-resonance frequency using an LCR meter KEYSIGHT^®^, type E4980A. The net impedance *Z* and phase shift φ between the supplying voltage and the input current were measured for the frequency varying from 20 Hz to 500 kHz with a frequency step of 10 kHz. From the results presented in Figure 13, it is clear that the self-resonance frequency (*f*_crit_) fulfills the requirements given in Table 1.

**Figure 14:**
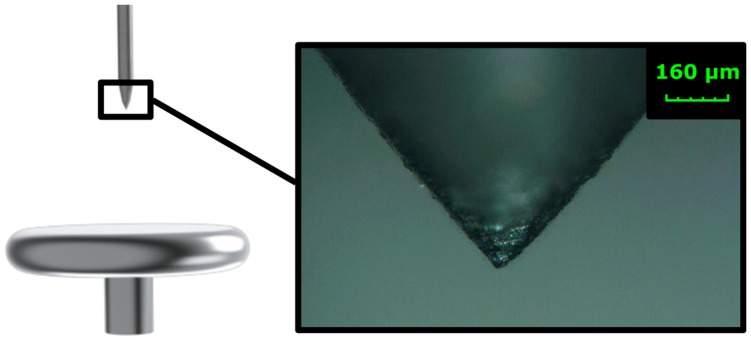
Experimental arrangement with a microscopic snapshot of the tip shape.

In order to demonstrate the influence of the filter, the basic corona PD test was performed [29]. The classical test needle-plane arrangement (Figure 14) was used for this purpose. The high voltage potential was applied at the tip and the plate was grounded. The distance between the metallic plane and tip was set to 40 mm. The tip radius is approximately 20 µm.

The experimental PD measurement was carried out according to standard IEC 60270 [21] and was connected as shown in Figure 1. The measurement circuit consists of a “PD-free” HV source (≤200 kV AC), a coupling capacitor (*C_k_* = 1000 pF ± 10%), a measuring impedance (LDM-5/U, *Z* = 50 Ω), and Partial Discharge Analyzer PD-SMART. The background noise was lower than 1.5 pC during the whole testing period.

**Figure 15:**
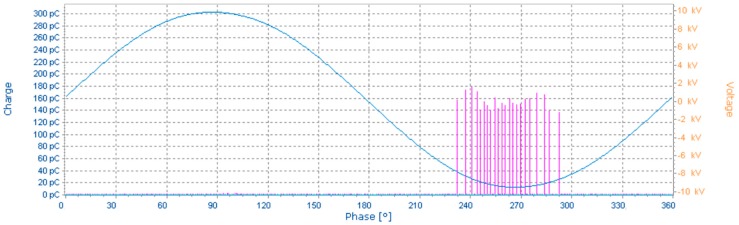
Oscillogram for 7 kV, needle-plane arrangement.

**Figure 16:**
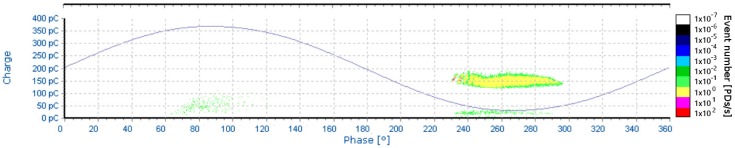
Pattern diagram for 7 kV, needle-plane arrangement.

The applied testing voltage (7 kV) corresponds to the voltage at which the first PD begin to appear. This voltage level is usually known as an inception voltage (PDIV). Typical oscillogram and pattern diagrams recorded during the measurement are shown in Figure 15 and Figure 16.

**Figure 17:**
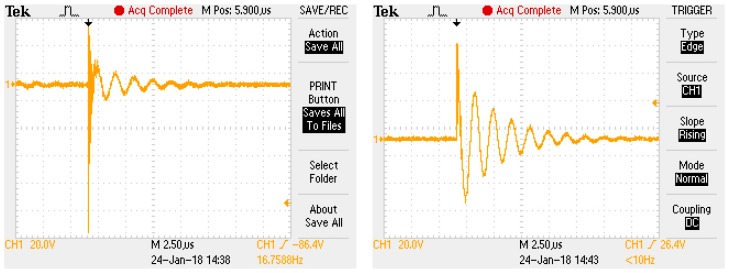
PD signal without filter (**left**); PD signal with filter (**right**).

The analyzed signal was measured with a digital oscilloscope Tektronix TDS 2024B. In Figure 17, the left-hand side presents the individual pulse shape measured without the filter. As compared to the right-hand side, which describes the same pulse, but measured with the filter, it includes some high frequency (HF) distortion, which deforms its shape.

**Figure 18:**
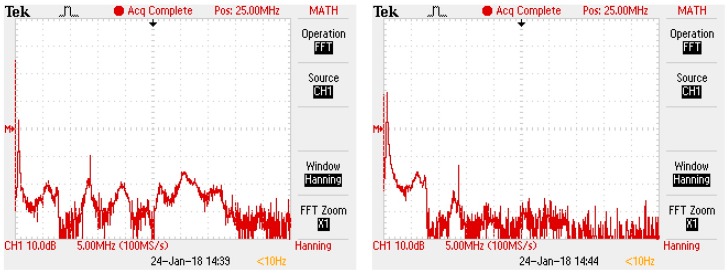
FFT of PD signal without filter (**left**); FFT of PD signal with filter (**right**).

This deformation also influences the frequency domain of the signal (see Figure 18). The fast Fourier transformation (FFT) analyses of the relevant waveforms (left—without filter, right—with filter) demonstrate that the filter can block higher frequencies that may possibly cause the interference.

## 6. Conclusions

With respect to the simulation data and design requirements, the experimental results indicate relatively sufficient accuracy. The proposed analytical approach quickly calculates fundamental dimensions together with an initial overview of the coil’s construction layout. The introduced thermal model presents the ability to predict the temperature anywhere inside the winding. It can be adopted for any similar geometry and can be used for any electrical device.

As was shown, with the careful design of the winding, we can significantly reduce the stray capacitance of the filter, which consequently increases the quality factor. Or, the design process could be used to manipulate the value of stray capacitance in order to reach a specified self-resonance frequency range of the filter.

## Figures and Tables

**Table 1:** Technical parameters required for the filter.

Parameter	Value
Self-inductance	*L* = 6 [mH]
Rated current	*I* = 8 [A] (AC, 50/60 Hz, sinus wave)
Current density	*J* < 3 [A/mm^2^]
Ambient temperature	*ϑ*_a_ = +40 [°C]
Self-resonance frequency range	*f*_crit_ = 80 ÷ 140 [kHz]

**Table 2:** Important design and construction properties.

Parameter	Value
Wire diameter	1.9 [mm]
Number of turns per layer	18
Number of layers	12
Total number of turns	216
*d* |*s*| *l* (Figure 3)	82 [mm] | 24 [mm] | 36 [mm]
Mass	2.5 [kg]

**Table 3:** Stray capacitance matrix calculated for transposed winding (Figure 9—left, blue).

[pF]	*C*_1_	*C*_2_	*C*_3_	*C*_4_	*C*_5_	*C*_n_
***C*_1_**	53	24.5	25.2	0.3	2	
***C*_2_**	24.5	76	2.1	24.3	22.7	
***C*_3_**	25.2	2.1	78	0.02	23.2	
***C*_4_**	0.3	24.3	0.02	76	2	
***C*_5_**	2	22.7	23.2	2	101	
***C*_n_**						
